# α-Hemihydrate calcium sulfate/n-hydroxyapatite combined with metformin promotes osteogenesis *in vitro* and *in vivo*

**DOI:** 10.3389/fbioe.2022.899157

**Published:** 2022-09-30

**Authors:** Sirui Liu, Haojie Fu, Yan Lv, Jing Jiao, Runying Guo, Yanyu Yang, Wenhang Dong, Hongyan Mi, Meiyue Wang, Mengzhe Liu, Rui Li

**Affiliations:** ^1^ Department of Stomatology, First Affiliated Hospital of Zhengzhou University, Zhengzhou, China; ^2^ Beijing Institute of Dental Research, Beijing Stomatological Hospital, Capital Medical University, Beijing, China; ^3^ College of Materials Science and Engineering, Zhengzhou University Zhengzhou, Zhengzhou, Henan, China; ^4^ Department of Stomatology, Beijing Friendship Hospital, Capital Medical University, Beijing, China

**Keywords:** metformin, osteogenic differentiation, BSP, RUNX2, Opn1

## Abstract

This study aimed to examine the effects of loading different concentrations of metformin onto an α-hemihydrate calcium sulfate/nano-hydroxyapatite (α-CSH/nHA) composite. The material characteristics, biocompatibility, and bone formation were compared as functions of the metformin concentration. X-ray diffraction results indicated that the metformin loading had little influence on the phase composition of the composite. The hemolytic potential of the composite was found to be low, and a CCK-8 assay revealed only weak cytotoxicity. However, the metformin-loaded composite was found to enhance the osteogenic ability of MC3T3-E1 cells, as revealed by alkaline phosphate and alizarin red staining, real-time PCR, and western blotting, and the optimal amount was 500 µM. RNA sequencing results also showed that the composite material increased the expression of osteogenic-related genes. Cranial bone lacks muscle tissue, and the low blood supply leads to poor bone regeneration. As most mammalian cranial and maxillofacial bones are membranous and of similar embryonic origin, the rat cranial defect model has become an ideal animal model for *in vivo* experiments in bone tissue engineering. Thus, we introduced a rat cranial defect with a diameter of 5 mm as an experimental defect model. Micro-computed tomography, hematoxylin and eosin staining, Masson staining, and immunohistochemical staining were used to determine the effectiveness of the composite as a scaffold in a rat skull defect model. The composite material loaded with 500 µM of metformin had the strongest osteoinduction ability under these conditions. These results are promising for the development of new methods for repairing craniofacial bone defects.

## Introduction

The regeneration of bone-tissue defects caused by trauma, congenital malformation, or tumor resection has long been a very challenging clinical problem. Physical and psychological conditions caused by bone-tissue defects may potentially be fatal (Cui et al., 2018; Borgiani et al., 2021). Tissue engineering can be used to repair these defects, producing results that more closely resemble the physiological state and avoiding some of the complications of conventional bone transplantation (Zhao et al., 2021; Fillingham and Jacobs, 2016). The three key components of tissue engineering are seed cells, induced microenvironments, and scaffold materials (Fillingham and Jacobs, 2016; Zimmermann and Moghaddam, 2011), among which the scaffold materials are considered to be the most important. Calcium sulfate can be used as a regenerative material to repair bone defects, as it releases calcium ions in the surrounding microenvironment and promotes repair (Zimmermann and Moghaddam, 2011). However, at present, the clinical application of α-calcium sulfate (α-CSH) is beset with many problems, including rapid degradation. To overcome these problems, we added nano-hydroxyapatite (nHA) to α-CSH to hinder degradation. However, α-CSH/nHA materials may lead to an inflammatory environment at the material implantation site due to local heat production (Carinci et al., 2004). Immunohistochemical tests revealed that the implants contained low levels of tumor necrosis factor-α (TNF-α) and IL-6, which may have been an effect of the implants, inhibiting their ability to heal the defect. (Supplementary Figure S2A). Therefore, in this study, we attempted to use a drug to inhibit the weak inflammatory state caused by material implantation in animals and promote bone healing. However, although the anti-inflammatory drugs currently used clinically are effective, they cannot promote the healing of bone tissue. In 1998, a paper published in *The Lancet* recommended the use of metformin as a hydrophilic drug (Author Anonymous, 1998; Del et al., 2021; Ludvik et al., 2021), which has since become widely accepted as a first-line treatment for patients with type-2 diabetes mellitus. Many studies have shown that treatment with metformin can reduce the risk of bone fracture in diabetes patients (Fillingham and Jacobs, 2016; Oh and Song, 2020), and a recent retrospective study and meta-analysis further verified this conclusion (Katge and Patil, 2017). In addition, research has demonstrated that metformin can improve the bone-marrow microenvironment to some extent (Wu et al., 2016). Therefore, this study prepared metformin (Met)/α-CSH/nHA scaffolds by adding metformin in different concentrations to our originalα-CSH/nHA scaffolds, and the effects on osteogenic gene and protein expression were investigated. RNA sequencing was used to study changes in the mechanism of the osteogenesis process induced by this material. Finally, the ability of the Met/α-CSH/nHA scaffold to promote osteogenic healing was verified through a rat skull defect model.

## Materials and methods

### Preparation of met/α-CSH/nHA

First, we prepared α-CSH by thoroughly mixing 100 g of calcium sulfate dihydrate (Sigma) in 10 ml of pure water with stirring. Then, we placed the mixture into a closed vessel at a pressure of 0.13 MPa, thermostatically heated it to 123°C, and placed it in a dryer at 120°C. Finally, it was cooled to 25°C and ground thoroughly (Mao et al., 2013). Subsequently, we completely mixed this α-CSH with nHA. To ensure the homogeneity of the mixture, we first prepared a suspension by dissolving 1.2 g of α-CSH in anhydrous ethanol, which we subsequently mixed well with 0.8 g of nHA. After complete mixing, the suspension was dried in an electric ventilated drying oven at 65°C, yielding a mixture of α-CSH and nHA with a mass ratio of 5:4. To load α-CSH/nHA with different concentrations of metformin, different amounts of metformin (0, 250, 500, and 1,000 μM) were dissolved in pure water and mixed with the α-CSH/nHA powder to obtain Met/α-CSH/nHA. Subsequently, cylinders with a diameter of 5 mm and a height of 1 cm (Supplementary Figures S1Aa) were fabricated for *in vitro* experiments, whereas disks with a diameter of 5 mm and a height of 1 mm (Supplementary Figures S1Ab) were made for *in vivo* experiments, as shown in the Supplementary Figure S1A. In addition, we incubated the Met/α-CSH/nHA material with different concentrations of metformin in a 37°C incubator for 3 d at a mass ratio of 1:5 in a normal medium to obtain the material extracts.

### Characterization of Met/α-CSH/nHA

The surface morphology and structure of the material were observed using SEM (Zeiss Sigma 300), as shown in previous literature (Li et al., 2011). α-CSH, nHA, metformin, and α-CSH/nHA cylindrical materials loaded with different amounts of metformin (0, 250, 500, and 1,000 μM) were also separately ground for FTIR detection (STA8000-Frontier, Netherlands) at a scanning frequency of 32 times/s over a wavenumber range of 4,000–500 cm^−1^. The changes in functional groups were qualitatively examined based on differences in the infrared absorption spectra. The assay data were analyzed and plotted using Origin 8.0 software. To further characterize the vigilant structure of the material surface, XRD was carried out. The specific assay was performed as described in a previous report (Tong et al., 2022). The XRD parameters were set to a voltage of 40 kV, a current of 40 mA, continuous scanning over a 2θ range of 10–80°, a scan speed of 0.75°/s, and θ/2θ scan mode. These data were also analyzed and plotted in Origin 8.0.

### Degradation experiments and the release of metformin

*In vitro* immersion experiments were carried out in simulated body fluid (SBF) for α-CSH, nHA alone, and four Met/α-CSH/nHA composites with different metformin concentrations. Cylinders with a diameter of 5 mm and a height of 1 cm were prepared from each of the five materials, dried uniformly, and polished smooth. For each of the five materials, ten specimens were prepared for testing. Each test sample was labeled and weighed accurately to obtain the initial weight (M1). The samples were immersed in a centrifuge tube containing 5 ml of SBF. The samples were placed on a shaker (at 5 rpm) at 37°C for *in vitro* degradation experiments. The SBF was changed every 7 d. After vacuum drying for 24 h, the degraded sample was weighed accurately to obtain weight M2. The degradation rate was calculated as a weight loss, i.e., (M1—M2)/M1. The weights of the three samples were averaged, and the weight loss versus time curve was plotted.

Since metformin cannot be accurately measured at lower concentrations, to study the ability of metformin release from this scaffold material, we prepared about 0.25 g of /α-CSH/nHA composite containing 1,000 um metformin (diameter of 3 mm and a height of 5 mm), and using a UV/VIS Spectrometer (Lambda 35, PerkinElmer) to measure the release of metformin, as shown in the previous literature (Leng et al., 2022). Briefly, it was placed in approximately 2 ml of SBF solution, and the supernatant was taken at 1w, 2w, and 3w for measurement.

### CCK-8 and EdU assay for the cytotoxicity of met/α-CSH/nHA

MC3T3-E1 cells are mouse cranial osteogenic precursor cells. MC3T3 was cultured using 10% fetal bovine serum in α-MEM; the fluid was changed every 2 d. MC3T3-E1 cells were inoculated into a 96-well plate and incubated with extracts of the Met/α-CSH/nHA scaffold containing different concentrations of metformin (0, 250, 500, or 1,000 μM) for 1–5 d. Then we use Cell Counting Kit 8 (CCK-8) (Dojindo, Japan) to detect the affection of this material on cell proliferation. Subsequently, we used EdU to assay the effect of different loading amounts of metformin in the Met/α-CSH/nHA material extracts on cell proliferation activity, as shown in a previous stud (Gong et al., 2020). To further validate the biocompatibility of the scaffold material, We resuspended MC3T3-E1 cell suspension on the surface of each group of scaffold material. After 48 h of incubation, the cells on their surface were digested using EDTA-free trypsin and stained using Tepant Blue to study cell survival.

### Hemolysis assay for the biocompatibility of met/α-CSH/nHA

Approximately 10 ml of venous blood extracted from the ear veins of three New Zealand white rabbits was mixed with normal saline at a volume ratio of 1:1.25. Normal saline was used as the negative control treatment, pure water was used as the positive control treatment, and extracts from the Met/α-CSH/nHA scaffolds loaded with different amounts of metformin were used as the experimental treatments; in each case, approximately 2.5 ml of each extract was used for incubation for about 30 min. Then, 0.05 ml of the rabbit blood was added to each of the mixtures in centrifuge tubes. The tubes were then stored in a water bath box at 37°C for 60 min. After centrifugation, 50 μL of the supernatant was extracted, and its absorbance was measured at 545 nm. The results were used to calculate the hemolysis rate of each treatment condition as follows: hemolysis rate (%) = (absorbance of the experimental group–absorbance of the distilled water group)/(absorbance of the normal saline group–absorbance of the distilled water group).

### Real-time PCR detection of changes in the osteogenic gene expression

MC3T3-E1 cells were inoculated into six-well plates. The extracts from the four different Met/α-CSH/nHA scaffolds (0, 250, 500, and 1,000 μM) were incubated with the cells for 7 d, and then, the total RNA was extracted from the digested cells. Real-time PCR was used to analyze the changes. The major genes included *Bsp*, *Opn*, and *Runx2*. The real-time PCR primers used are listed in Table 1 **.**

**TABLE 1:** Sequences of primers used for real-time PCR.

Gene	Forward primer	Reverse primer
*Runx2*	5′-GCT​TCT​CCA​ACC​CAC​GAA​TG	5′-GAA​CTG​ATA​GGA​CGC​TGA​CGA
*Bsp*	5′-AGA​GTG​ACG​GTG​TCG​TAG​CC	5′-TGT​AGG​TGC​TGT​GGT​CAA​GG
*Opn*	5′-CAT​GGC​TGG​TCT​TCC​CGT​TGC	5′-GAC​GGC​CGA​GGT​GAT​AGC​TT
*Gapdh*	5′-TGA​TTC​TAC​CCA​CGG​CAA​GTT	5′-GAG​ATG​ATG​ACC​CTT​TTG​GCT

### Western blot analysis to detect changes in osteoblast-related protein expression

Similar to the process described in *Real-time PCR detection of changes in the osteogenic gene expression* Section, MC3T3-E1 cells were incubated for 14 d. The cells were digested to extract protein, and a bicinchoninic (BCA) kit was used to determine the protein concentration. A loading buffer (5×) was added at a volume ratio of 4:1, and the protein was denatured by boiling at 100°C for 5 min; the protein sample size was 30 μg. Subsequently, polyacrylamide gel electrophoresis (SDS-PAGE), membrane transfer, sealing, incubation of primary antibodies (*Bsp*, *Opn*, and *Runx2*), incubation, and development of secondary antibodies were carried out sequentially.

### ARS and ALP staining

To further clarify the ability of each Met/α-CSH/nHA scaffold with different metformin concentrations (0, 250, 500, and 1,000 μM) to induce osteogenesis and mineralization, we cultured the induced cells for 14 d using extracts from each material.

We stained these cells with alizarin red stain (ARS, Cyagen). Subsequently, we added 1 ml of 1% cetyl pyridine solution to each well of the six-well plate for about 24 h. After completely dissolved, 100 μL was pipetted into the 96-well plate, and the solution measured the absorbance value at 490 nm.

Also, we stained these cells with alkaline phosphatase (BCIP/NBT Alkaline Phosphatase Color Development Kit, Beyotime) staining. To quantify the ability of each group of materials to induce osteogenic differentiation of cells. We performed measurements of alkaline phosphatase activity. The specific experimental methods were referred to in previous literature (Wang et al., 2020). Briefly, at 14 d, total protein was extracted using RIPA lysis buffer without adding phosphatase inhibitors, and 15 μg of each group were sampled. Alkaline phosphatase activity was assayed according to see alkaline phosphatase kit instructions.

### RNA-seq study of met/α-CSH/nHA-induced osteogenic mechanism of MC3T3-E1 cells

For this experiment, six samples were divided into a control group and Met/α-CSH/nHA scaffold group. RNA-sequencing technology (RNA-seq) was used to detect RNA sequences, as described elsewhere (Lybrand et al., 2019). The significance of the terms and pathways were computed as Q values, with a rigorous threshold (Q value ≤0.05), using Bonferroni corrections.

### Rat cranial defect-repair experiment

SD rats aged 6 weeks were selected for adaptive culture at the Experimental Animal Center of Zhengzhou University for approximately 1 week. Then, SD rats were injected intramuscularly with pentobarbital. Cranial defects with a diameter of approximately 5 mm were artificially prepared under aseptic conditions. The defects were filled with Met/α-CSH/nHA scaffolds with different metformin concentrations (0, 250, 500, and 1,000 μM). The specific experimental procedure is shown in Figure 4A. The animals were kept normally for 10 weeks after the operation. Every 2 d, their living environment was cleaned, and the bedding and water were changed. Ten weeks later, samples were detected by micro-computed tomography (micro-CT, Bruker-microCT, Belgium). To reduce material-induced bias in CT analysis, we defined the Micro-CT region of interest (ROI) as the area of regenerable new bone within 150 layers (2 mm) above the edge of the bone defect. Subsequently, bone volume/total volume (BV/TV), trabecular thickness (Tb.Th), trabecular separation (Tb.Sp), and trabecular number (Tb.N) in the ROI were statistically analyzed. (Jiang et al., 2020). Hematoxylin and eosin (H&E) staining, Masson staining, and immunohistochemical staining were employed. The results of the immunohistochemical staining were quantitatively analyzed by Seville image analysis system. The following indicators were analyzed: a positive area ratio (positive area ratio: positive area/tissue area) and Histochemistry score (H-SCORE = ∑(pi × i) = (percentage of weak intensity area x 1) + (percentage of moderate intensity area x 2) +(percentage of strong intensity area x 3), where, pi represents the percentage of the positive signal pixel area, and i represents the positive level. NRecon software (Bruker microCT) was then applied to reconstruct 3D images. Additionally, CTAn software (Skyscan, Konitch, Belgium) was used to process the data for analysis.

### Statistical analysis

The results were expressed as mean ± SD of three independent experiments. The data were analyzed using Prism6 software (Graphpad, USA), and statistical analyses were performed with the Student’s t-test. A *p*-value <0.05 was considered statistically significant.

## Results

### Characterization of Met/α-CSH/nHA scaffolds with different drug concentrations

**FIGURE 1:**
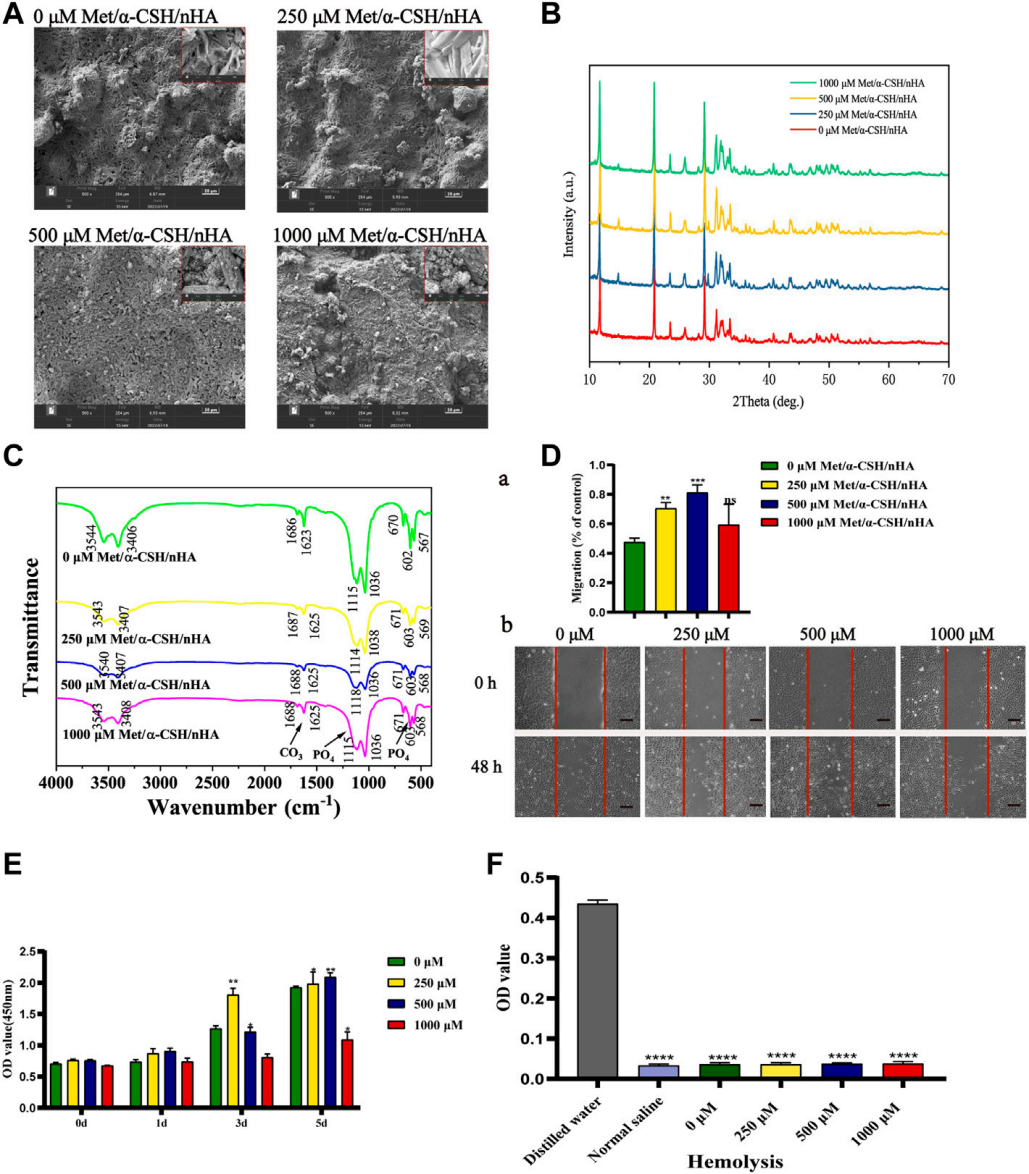
Characterization of Met/α-CSH/nHA prepared with different metformin concentrations (0, 250, 500, and 1,000 μM). SEM **(A)** (Scale bar = 20 μm), XRD **(B)**, FTIR **(C)** demonstrate that metformin is physically bound to α-CSH and nHA and can be stably present. Cell scratch assay **(D)** demonstrates that the material containing 500 μM of metformin is more capable of promoting cell migration (Scale bar = 200 μm); CCK8 **(E)** and haemolysis assay **(F)** show that all groups of materials are more biocompatible and less likely to cause tissue haemolysis.

We used SEM to characterize the surface morphology of the Met/α-CSH/nHA scaffolds. As shown in Figure 1A, the crystal, surface, and porous structures of the pristine materialsshowed that α-CSH/nHA were not significantly changed by the presence of metformin at different concentrations of 0, 250, 500, or 1,000 μM. The results of SEM of metformin, nHA and α-CSH are shown in Supplementary Figure S1B. XRD and FTIR were used to determine the internal elemental composition of the material. As shown in Figure 1B, XRD patterns included the characteristic crystalline diffraction peaks of α-CSH/nHA mainly at approximately 24–25° and 30–34°, and these peaks overlapped for all materials. Wide XRD peaks typically imply a lack of crystallinity. In addition, the apatite phase diffraction peak was observed at approximately 25.9°. The broad diffraction peak at 30–34° was assigned to hydroxyapatite (Valentim et al., 2018; Lybrand et al., 2019). The XRD results indicate that all the composites were composed of calcium sulfate and nHA. The XRD patterns of the samples with higher concentrations of metformin showed peaks with higher intensities. However, the diffraction angles of the peaks were the same for each material, indicating that adding metformin did not affect the structure of α-CSH/nHA or its related properties. The FTIR results of the composite materials are shown in Figure 1C. An absorption peak observed in the range of 3,542–3,574 cm^−1^ was assigned to calcium sulfate, and the peaks at 1,622–1,685 cm^−1^ correspond to the bending of the bond between hydrogen and oxygen, not bonds to these elements in general bonds. Peaks in the ranges 600–680 cm-1 and 1,110–1,150 cm-1 correspond to the stretching and bending of the S-O bond, respectively (Ren et al., 2022). The results of XRD and FTIR of metformin, hydroxyapatite and α-CSH are shown in Supplementary Figures S1C,D. The peak intensities and spectral positions did not change noticeably with the metformin concentration.

To further study the biocompatibility of the composite material, we performed cell scratch, CCK-8, EdU, and hemolysis assays. As shown in Figure 1D, the cell-scratch test results indicated that the composite extracts prepared using 250 μM of metformin promoted cell migration, in contrast with the control treatment without metformin. In contrast, the 500 and 1,000 μM metformin treatments had little effect on cell healing. In addition, in the CCK-8 (Figure 1E), lower metformin concentrations promoted cell proliferation; cell proliferation was induced within 3–5 d. However, in the 1,000 μM group, a more apparent inhibitory effect on cell proliferation was observed. In the Edu assay (Supplementary Figure S1H), The ratio of EdU-positive cells to the total number of cells alive (the proportion of cells in the proliferative phase) was not significantly different between scaffold materials with lower metformin concentrations (below 500 μM) and those without metformin addition, while higher concentrations of metformin (1,000 μM) reduced cells in the proliferative phase, and the difference was statistically significant. We extracted blood from New Zealand white rabbits for hemolysis assays to further clarify the material’s biocompatibility. The measured absorbances of the extracts of α-CSH/nHA loaded with metformin at various concentrations are shown in Figure 1F and Table 2. In addition, growth experiments with cells loaded directly onto the surface of the scaffold material have demonstrated that the material has no significant inhibitory effect on cell activity (Supplementary Figure S1G). The results indicated a low hemolytic potential for the materials. Hence, the composite materials were considered to have good biocompatibility.

**TABLE 2:** OD values of Met/α-CSH/nHA with different metformin concentrations.

Groups	Od value	Hemolysis rate
Distilled water	0.4351 ± 0.005413	—
Normal saline	0.03361 ± 0.001628	—
0 μM	0.03599 ± 0.002118	0.0059272
250 μM	0.03618 ± 0.002088	0.064041
500 μM	0.03770 ± 0.0009249	0.0101753
1,000 μM	0.03795 ± 0.002562	0.0107985

It takes 3–6 months for bone trabeculae to be reconstructed at the site of the bone tissue defect (Garcia et al., 2022). To study the stability of the met/α-CSH/nHA materials, degradation, and metformin release experiments were carried out. The results are shown in Supplementary Figure S1E, where the weight loss rate of each group of scaffold material gradually increased over time. All groups of stent material showed material degradation in the first week. Of these, the nHA group degraded more rapidly, with a weight loss ratio of around 25% at 1 week. Furthermore, the WLR curves of the α-CSH/nHA materials containing different concentrations of metformin showed the same trend of slowly increasing at 4 weeks. This indicates that the concentration of metformin has a negligible effect on the degradation of α-CSH/nHA. Meanwhile, in the 0 μM Met/α-CSH/nHA, a small amount of material remnants were visible on CT findings at 10 weeks (Supplementary Figures S1Eb). These indicate that the material has good biodegradability and can provide support to the defect area. Modulating the slow release of drugs is an effective way to prolong the duration of drug action (Leng et al., 2022). We found that Met/α-CSH/nHA scaffold material loaded with 1,000 μM shows a more substantial release (about 20%) within the first week, which can be sustained for around 2–3 weeks (Supplementary Figure S1F). The duration of action of metformin loaded in α-CSH/nHA scaffold material is considerably longer compared to direct metformin use. Therefore, these results suggest that met/α-CSH/nHA scaffold material has good biocompatibility and metformin controlled release ability and can be applied as a promising scaffold material.

### Effects of met/α-CSH/nHA on MC3T3-E1 cell osteogenic capacity

**FIGURE 2:**
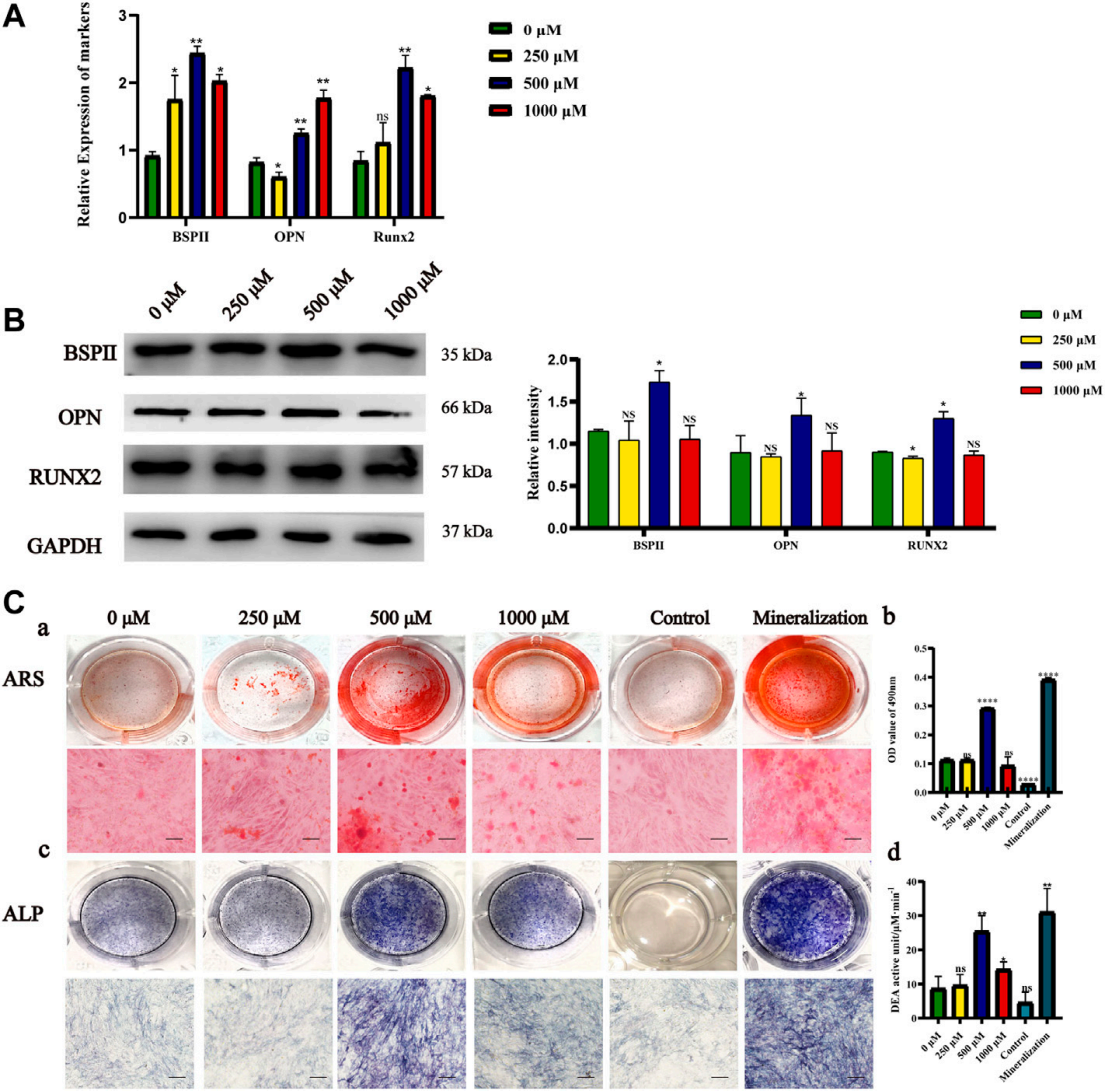
Osteogenic capacity effects of Met/α-CSH/nHA materials prepared with different metformin concentrations (0, 250, 500, and 1,000 μM). The expression of Osteogenic-specific mRNA and protein was assessed by qRT-PCR **(A)** and Western Blot **(B)**. ARS results **(Ca,b)** (Scale bar = 50 μm), ALP staining **(Cc)** and the results of phosphatase activity assay **(Cd)** are consistent with Western blot results **(B)**.*p < 0.05 and **p < 0.01 compared with the 0 μM group.

We incubated MC3T3-E1 cells with Met/α-CSH/nHA extracts and investigated their effects on the osteogenic ability of the cells by real-time PCR and western blotting. As shown in Figures 2A, 500 μM Met/α-CSH/nHA material induced significantly higher expression of BSP and RUNX2 at 7 d than the group with concentrations lower than 500 μM and the group with 1,000 μM, while the expression of OPN was highest in the group with 1,000 μM. Runt-associated transcription factor 2 is a crucial transcription factor in osteoblastogenesis and controls the production of type I collagen, osteochondroitin (OPN), osteosalivary protein, osteocalcin, and fibronectin (Thant et al., 2022). OPN is mostly expressed in mineralized tissues, hence its steady expression may be a little later than that of RUNX2 (Vancea et al., 2021). Additionally, we used the Western Blot to analyze the expression of RUNX2 at 7 days, and the findings revealed no discernible variations in that expression (Supplementary Figure S2B). We next looked at the expression of BSP, OPN, and RUNX2 at 14 days and discovered that these osteogenesis-related proteins were considerably greater at 500 μM Met/-CSH/nHA than in the other groups at 14 d (Figure 2B). In summary, the composite loaded with 500 μM metformin had the strongest ability to induce osteogenesis, while those of the composites loaded with 250 and 1,000 μM metformin were enhanced to some extent relative to that of the control but were weaker than that of the former. The results of staining with ALP and ARS and the phosphatase activity assay supported this conclusion (Figure 2C). ARS (Figures 2Ca,b), ALP staining (Figure 2Cc), and phosphatase activity assay (Figure 2Cd) showed that the scaffold material extract loaded with 500 μM metformin induced osteogenic differentiation of cells similar to that of the osteogenic induction solution and was significantly higher than that of the control group.

### RNA-seq study of the mechanism of met/α-CSH/nHA effect on MC3T3-E1 cell osteogenic ability

**FIGURE 3:**
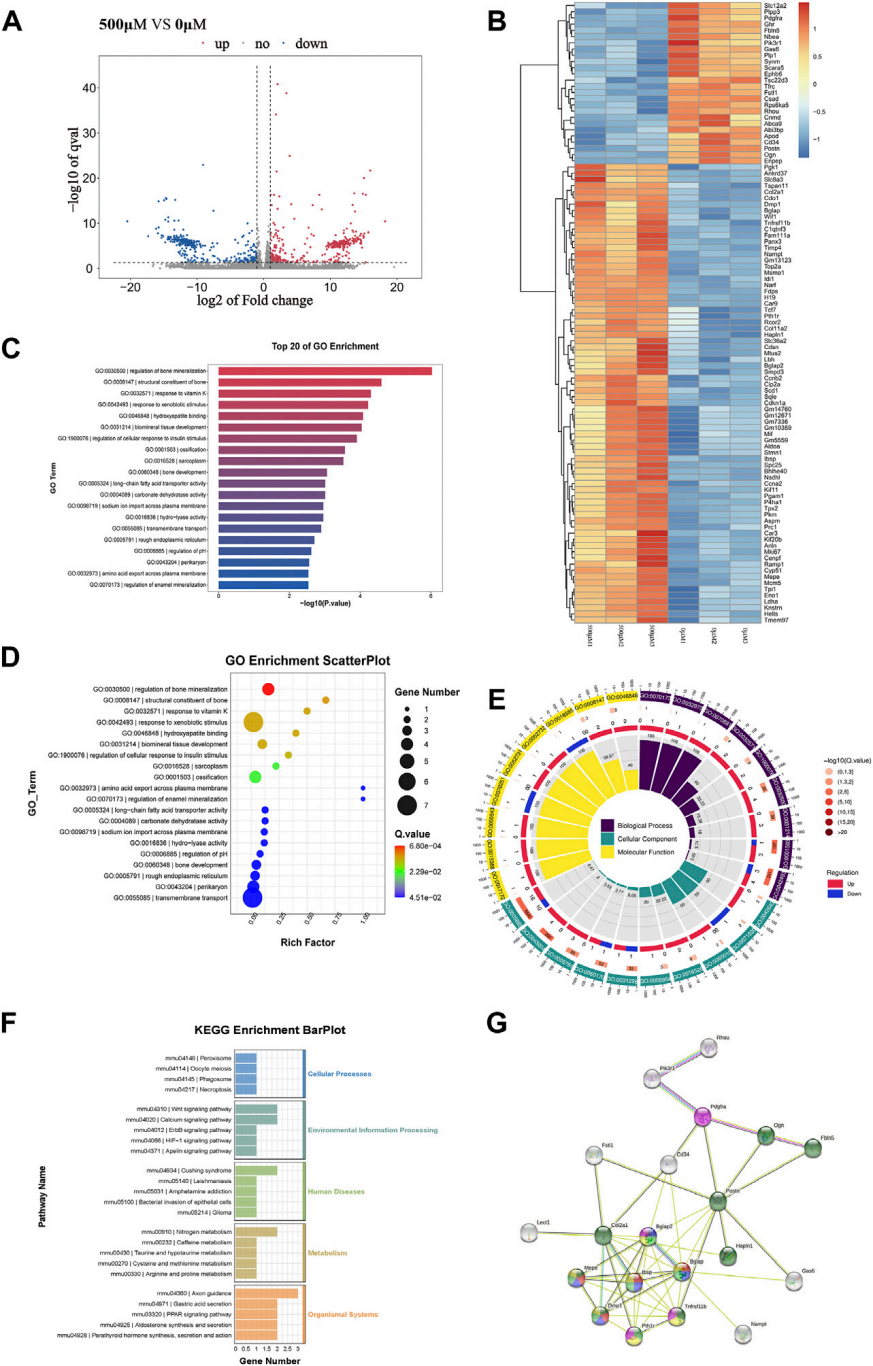
The results of RNAseq between 0 μM Met/α-CSH/nHA and 500 μM Met/α-CSH/nHA.The differentially expressed genes between 0 μM group and 500 μM are shown in volcano **(A)** and heatmap **(B)**. GO Scatterplot, top 20 of GO enrichment and GO loopcircos are shown in **(C–E)**. KEGG enrichiment barplot is shown in **(F)**. The correlation of protein interactions predicted by differential protein is shown in **(G)**.

To further investigate the mechanism underlying the MC3T3-E1 osteogenesis induced by the composite materials, RNA-seq was used to sequence the cells after induction and in the control group. As shown in Figure 2, the composite loaded with 500 μM metformin had the strongest ability to induce MC3T3-E1 osteogenesis. Therefore, 500 μM metformin was selected as the induction group. The upregulated and downregulated genes for the cells induced by the composite materials with 0 and 500 μM metformin are shown in Figure 3A. The heat map of the 100 most significantly different genes is shown in Figure 3B. Gene Ontology (GO) is an internationally standardized functional classification system for genes that comprehensively describes the properties of genes and their products in any organism (Ashburner et al., 2000). The top 20 of GO enrichment are shown in Figure 3C. The most significantly altered product obtained by enrichment is associated with bone mineralization and formation. In addition, the color of the GO enrichment map reflects the degree of enrichment of the differential genes in the GO term, with the darker the color, the more significant the enrichment (Gene Ontology Consortium, 2021). GO bubble charts lead to consistent conclusions (Figure 3D). Figure 3E shows the GO loopcircos diagram. The first circle (from outside to inside) shows the GO entries enriched for Top (smallest *p*-value or smallest Q-value). Red represents up-regulated, blue represents down-regulated, the number indicates the number; the fourth circle indicates the percentage of enrichment factors (Rich. Factor). This loop suggests that the scaffold material containing 500 μM may affect cell differentiation (Biological Process) by affecting the binding to the cellular protein and thus the binding to the associated protein (Molecular Function). We performed a KEGG enrichment analysis to explore the mechanism (Figure 3F) further. The results suggest that the induction may mediate the Wnt signaling pathway, calcium signaling pathway, or nitrogen metabolism. In addition, the results showed that the induction might cause changes in axon guidance and, at the same time, may be associated with Cushing syndrome.

To further explore the mechanism underlying changes in the osteogenesis process of the MC3T3-E1 cells as induced by the composite materials, the STRING database (https://string-db.org/) was used to establish a network enrichment analysis for the abovementioned 100 differentially expressed genes (Figure 3B). Subsequently, we selected sets with >3 proteins with reciprocal relationships. The protein interactions are shown in Figure 3G. Of note, markers associated with extracellular matrix are green; those associated with enamel mineralization and bone regulatory proteins are red; those associated with osteocalcin and enamel mineralization are purple; those associated with biomineralization and osteogenic signaling are yellow; and those associated with only ostogenic signaling are pink. The results showed that the number of nodes is 29; the number of edges is 49; and the PPI enrichment *p*-value:< 1.0e-16, indicated that the result is statistically significant. Developing chondrocytes that are actively multiplying and have a high level of Col2a1 expression. Additionally, Runx2 promotes osteoblast maturation in immature osteoblasts by regulating the expression of bone matrix protein genes, such as Col1a1, Col1a2, Spp1, Ibsp, and bone carboxyglutamate protein (Bglap)/Bglap2. Apatite crystals also require osteocalcin (Bglap/Bglap2) expression, which is parallel to the alignment of collagen fibrils (Komori, 2022). Our reciprocal plots and GO, KEGG enrichment findings indicate that 500 μM Met/-CSH/nHA may modulate the Wnt signaling pathway by controlling Runx2 expression to control osteoblast development as well as matrix protein gene expression in chondrocytes.

### SD rat skull defect model to verify the osteogenic ability of met/α-CSH/nHA

**FIGURE 4:**
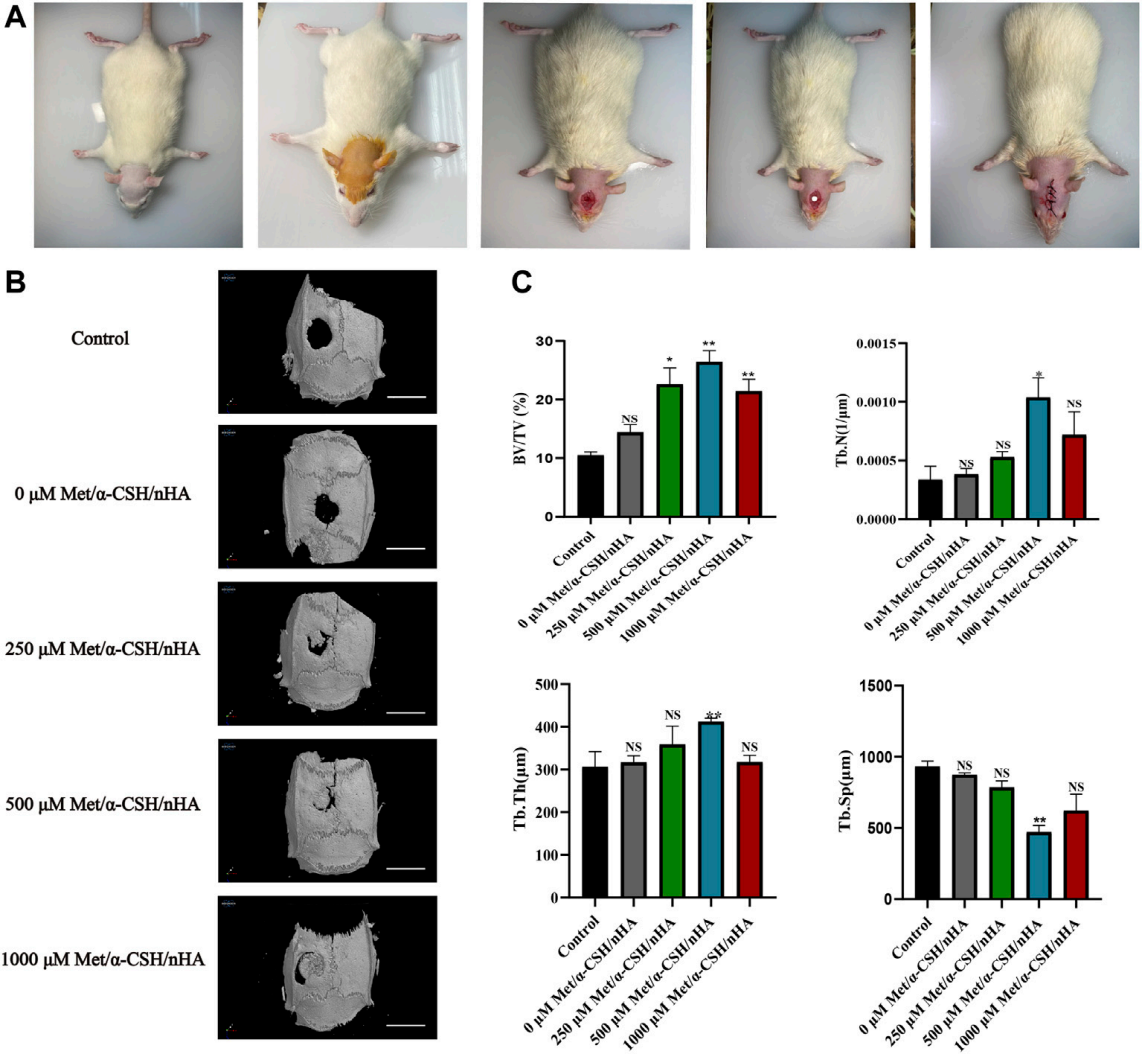
Preparation of SD rat skull defect model and micro-CT results after 10 weeks. The preparation of the rat cranial defect is shown in **(A)**. The micro-CT demonstrates the repair of the cranial defect at 10 weeks **(B)** (Scale bar = 5 mm) and was statistically analysed **(C)**.*p < 0.05 and **p < 0.01 compared with the control group.

Ten weeks after the faults were patched with Met/-CSH/nHA material, the rats’ skulls were taken out (Figure 4A). The Met/-CSH/nHA material had a strong capability to repair bone defects, and this ability was dependent on the concentration of metformin, according to the micro-CT data (Figure 4B). As shown in Figure 4C, the composite material could promote bone healing in rats in a concentration-dependent manner. As the metformin concentration increased to 500 μM, the bone volume fraction (BV/TV), trabecular number (Tb.N), and trabecular thickness (Tb.Th) in the region of interest all increased gradually, whereas the intertrabecular space (Tb.Sp) decreased. BV/TV, Tb. Tn, and Tb.N are positively correlated with bone healing, whereas Tb. Sp is negatively correlated with bone healing. Our *in vitro* findings and these outcomes are mutually exclusive.

**FIGURE 5:**
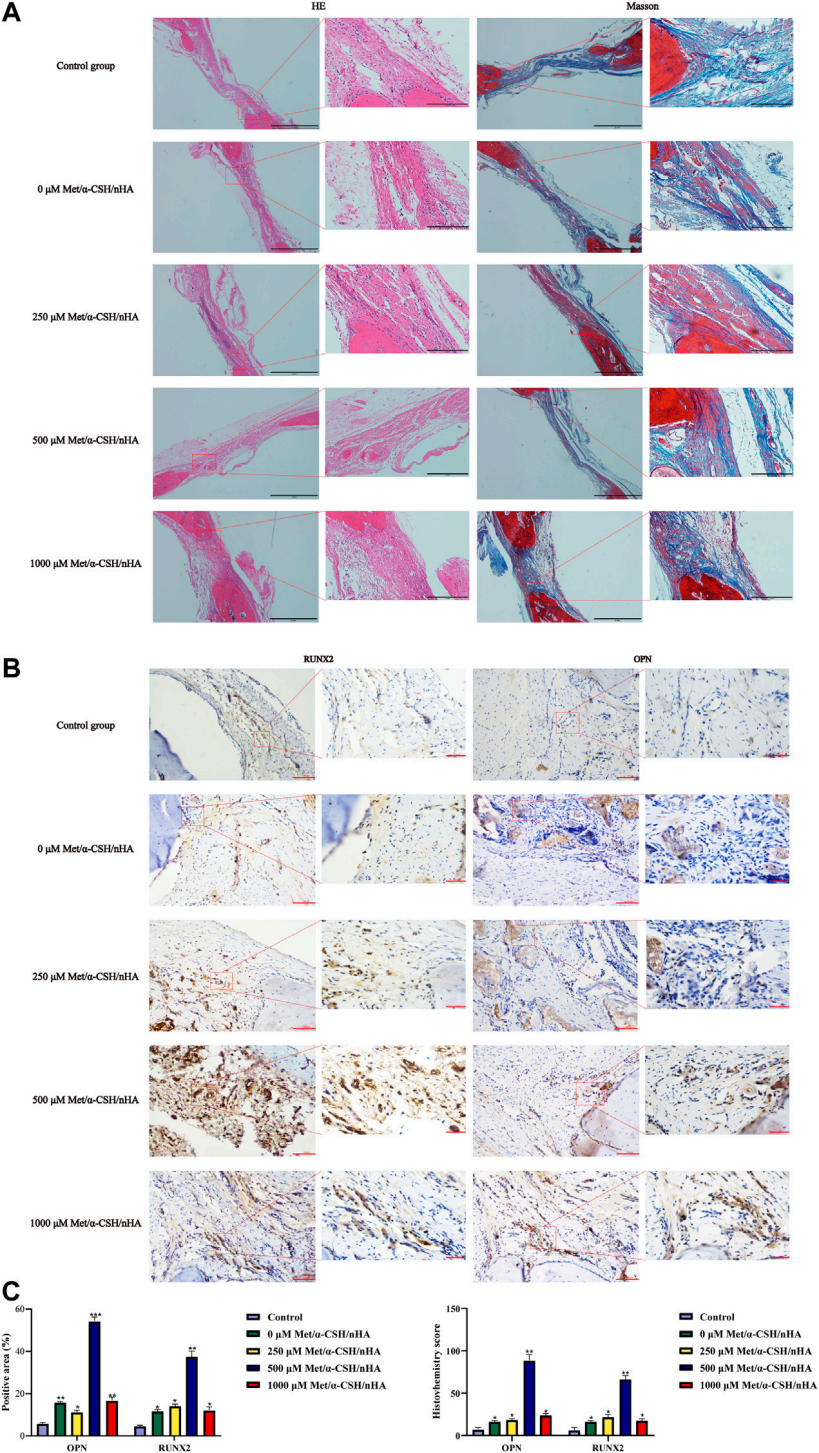
Met/α-CSH/nHA with different drug concentrations (0, 250, 500, and 1,000 µM) for enhancing osteogenic properties. The results of HE and Masson staining are shown in **(A)** (Scale bar = 2 mm). The expression of the osteogenesis-related genes RUNX2 and OPN is shown in **(B)** (Scale bar = 100 μm). The quantitative histochemical analysis **(C)** was based the positive area and H-SCORE.

To further investigate the characteristics and composition of the new tissues, after 10 weeks, we stained the specimens with H&E and Masson’s trichrome. The trabecular bone is an extension of the bone cortex in the cancellous bone (Shi et al., 2020). As shown in Figure 5, H&E staining revealed that the repair site was completely surrounded by soft tissue in the control group, whereas it was almost completely surrounded by collagen tissue, which appears blue in Masson staining. As the metformin concentration increased to 500 μM,, the formation of bone trabeculae at the repaired site of the composite material increased, and no soft tissue was observed. Masson staining revealed the formation of concentric rings resembling osteogenic nodules in the 500 μM Met/α-CSH/nHA material, which was marked by mature osteogenic tissue formation appearing red. Consistent with the results of the *in vitro* experiments, the osteogenic effect of the 1,000 μM Met/α-CSH/nHA treatment was weaker but still stronger than that of the control treatment (Figure 5B). Similarly, the quantitative statistical analysis along with histochemical staining (Figure 5C), with indices such as immunohistochemical indices and positive area immunohistochemical-specific staining results showed that expression of the Opn and Runx2 proteins gradually increased alongside bone healing as the metformin concentration increased, peaking when the metformin concentration was 500 μM. At 1,000 μM, this expression was slightly weaker but still stronger than that of the control treatment.

## Discussion

In recent years, bone-tissue engineering has become an indispensable technique for treating bone trauma. This approach provides a more efficient and less risky means to achieve autologous transplantation and allotransplantation (Hendrickx et al., 2021). In this investigation, we discovered that α-CSH/nHA composites that were metformin-loaded exhibited superior biocompatibility and could contribute in the repairing of significant cranial defects in SD rats. According to a related study, topical metformin use can enhance the biomechanical characteristics of the Achilles tendon and bone as well as the bone’s microstructure (Shi et al., 2022). Therefore, α-CSH/nHA composites loaded with metformin may play a therapeutic role in bone defects caused by infection, surgery, tumors, and trauma.

In this study, we started by analyzing the crystal structure and physical properties of α-CSH/nHA composites loaded with metformin using SEM, FTIR, and XRD. α-CSH/nHA exhibited typical α-CSH and nHA crystal morphology with large pores. The larger open pores provided the required nutrients for bone tissue regeneration and healing and oxygen transport conducive to osseointegration (Chen et al., 2018). FTIR and XRD have high sensitivity for analyzing the material composition, functional groups, and crystal structure (Venkatesan and Kim, 2014; Al et al., 2015). After adding metformin at various concentrations, it was discovered that the peaks of nHA and α-CSH were not considerably altered (Supplementary Figures S1C,D). It indicates that the substance was stable and that there was no chemical interaction between metformin and nHA or α-CSH.

Materials for bone restoration must be able to support cellular activity in order to be considered biocompatible (Bose et al., 2012; Gang et al., 2022). In this work, we used CCK-8, hemolysis assay, and EdU assay to further stimulate cells and confirm the material’s outstanding biocompatibility.

It has been demonstrated that metformin can protect bone microarchitecture, maintain bone density, and prevent bone loss, all of which are crucial for osteogenic differentiation (Leng et al., 2022). A related study incorporated metformin into a gelatin/nano-hydroxyapatite scaffold and found that the material was biocompatible, upregulated osteogenic protein expression and promoted alveolar bone regeneration in rats (Fang et al., 2022). This result is consistent with the results of the present study. However, we used a higher concentration of metformin (500 μM) compared to this study (50 μM). *In vitro* experiments, we demonstrated the concentration-dependent osteogenic induction of MC3T3 cells using qPCR, Western blot, ARS and ALP (Figure 2). *In vivo* experiments, The same concentration dependence of the scaffold material was found for the repair of critical bone defects in rat skull using HE staining, masson staining and immunohistochemical staining (OPN、RUNX2) (Figure 5). This effective concentration is in general agreement with the results of Huang et al. 500μM metformin promoted proliferation and osteogenic differentiation of MC3T3-E1 cells (Huang et al., 2022). For bone regeneration, activating the self-repair path that involves inhibition of inflammation, promoting osteogenesis, and creating a new vascular supply is essential (Zhao et al., 2022). The higher concentrations of metformin (1,000 μM) had some ability to induce osteogenic differentiation of cells. However, CCK-8 and EdU experiments found that the material extracts loaded with 1,000 μM metformin had a more vital ability to inhibit cell proliferation (Figure 1E, Supplementary Figure S1H). This may explain that the scaffold material loaded with metformin at 500 μM had the most vital ability to induce osteogenic differentiation of cells.

The regeneration process of bone tissue is regulated by multiple genetic signaling pathways (Ye et al., 2021). The Wnt/β-catenin signaling pathway plays an important role in cellular osteogenic differentiation (Ma et al., 2022; Wang et al., 2022). Metformin can activate Wnt/β-catenin signaling pathway and thus treat diabetic osteoporosis (Huang et al., 2022). In this study, the differential genes were enriched by RNAseq, and the GO enrichment results showed that the differential genes were more enriched in osteogenic mineralization and bone development, while the KEGG enrichment results showed that the genes related to Wnt signaling pathway and calcium signaling pathway were significantly expressed. In addition, the expression of fra-1(Fos-related antigen-1) in the canonical Wnt signaling pathway and CaMKII(calmodulin-dependent protein kinase II) in the Wnt/Ca^2+^ pathway were significantly higher (Supplementary Figure S2C). Additionally, we observed increased expression of RUNX2 after the stimulation of osteogenic differentiation utilizing 500 μM Met/α-CSH/nHA (Figures 2A,B). These results suggest that Met/α-CSH/nHA may mediate the expression of RUNX2 to affect canonical Wnt signaling pathway through regulation of fra-1 or affect the Wnt/Ca^2+^ pathway by influencing CaMKII expression to regulate bone tissue repair. The expression of osteoclast-related marker protein MMP-9 was reduced in Met/α-CSH/nHA containing 500 μM, suggesting that the osteogenic repair effect of this material may be related to the inhibitory effect on bone resorption (Supplenmentary Figure S2A).

## Conclusion

In this experiment, we developed a scaffold material loaded with metformin in α-CSH/nHA (Met/α-CSH/nHA). We verified the *in vitro* osteogenic ability of the materials using qPCR, Western blot, ALP, and ARS. In addition, the *in vivo* effect of the scaffold material on repairing bone defects was verified by preparing a rat cranial defect model and using the scaffold material for repair. These results provide a new theoretical and experimental basis for bone tissue engineering.

## Data Availability

The datasets presented in this study can be found in online repositories. The names of the repository/repositories and accession number(s) can be found in the article/Supplementary Material.
