# Supplementary material for: α-Hemihydrate calcium sulfate/n-hydroxyapatite combined with metformin promotes osteogenesis in vitro and in vivo
Source: Front Bioeng Biotechnol. 2022 Sep 30;10:899157. doi: 10.3389/fbioe.2022.899157 (PMC9563001; doi:10.3389/fbioe.2022.899157)
Supplement: Supplementary file 1 [file DataSheet1.pdf]

Supplementary Material

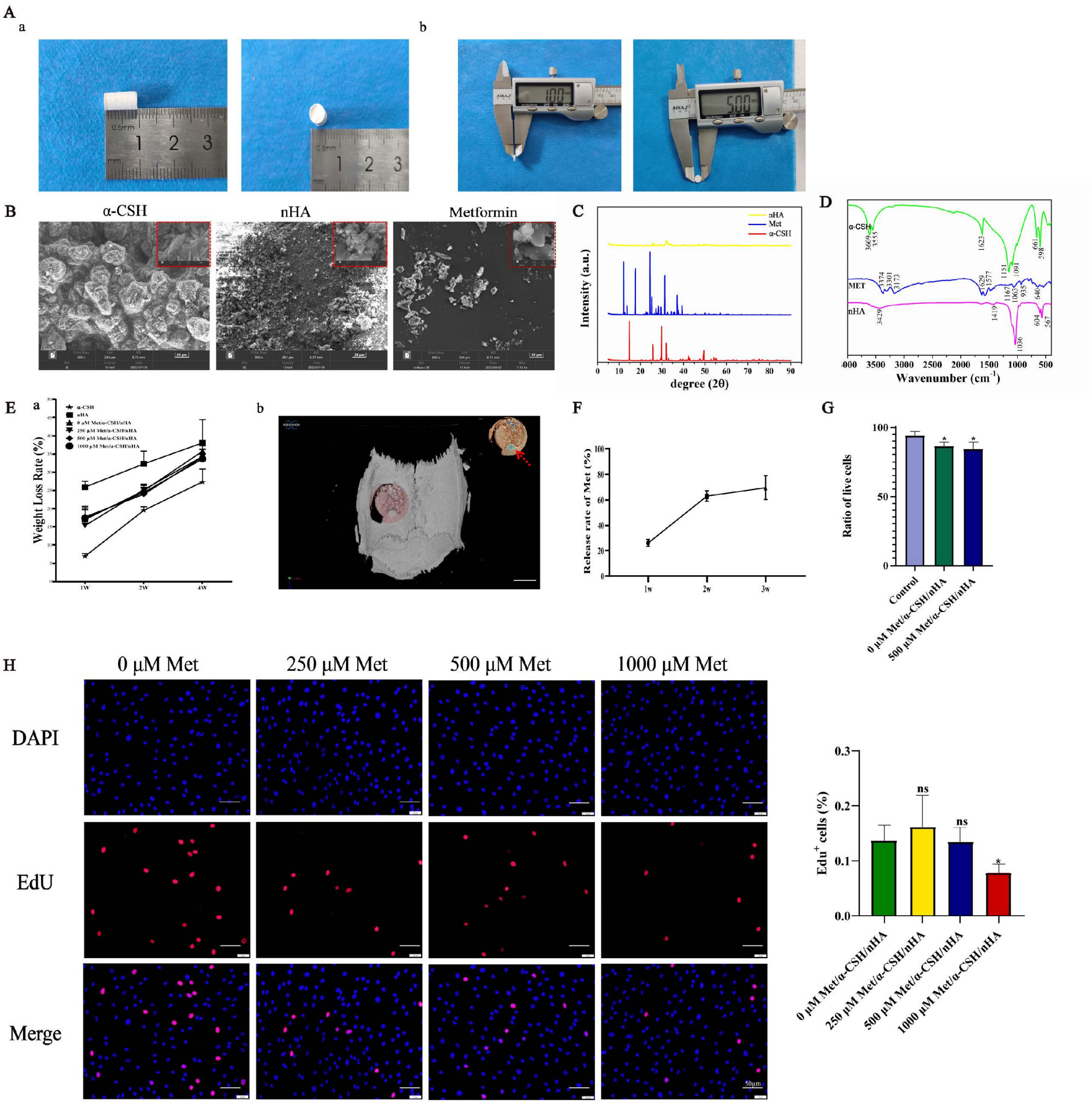

Supplementary Figure 1. Characterization of Met/α-CSH/nHA. The general view of the material is shown in A. SEM (B), XRD (C), and FTIR (D) show the surface structure, crystallographic analysis, and moiety composition of α-CSH, nHA, and Metformin, respectively. Ea plot shows the release profile of the weight loss rate of the material at different concentrations (0, 250, 500 and 1000 μM) of Met/α-CSH/nHA over four weeks. Eb plot shows the 1000 μM Met/α-CSH/nHA group after ten weeks. The arrows are the remaining material that is not completely degraded. F shows the percentage release of metformin from Met/α-CSH/nHA containing 1000 μM over three weeks.The survival rate of MC3T3-E1 cells grown directly on the material surface (G) and EdU experiments (H) further characterize the excellent biocompatibility of the material (Scale bar: 50 μm).

Supplementary Material

A

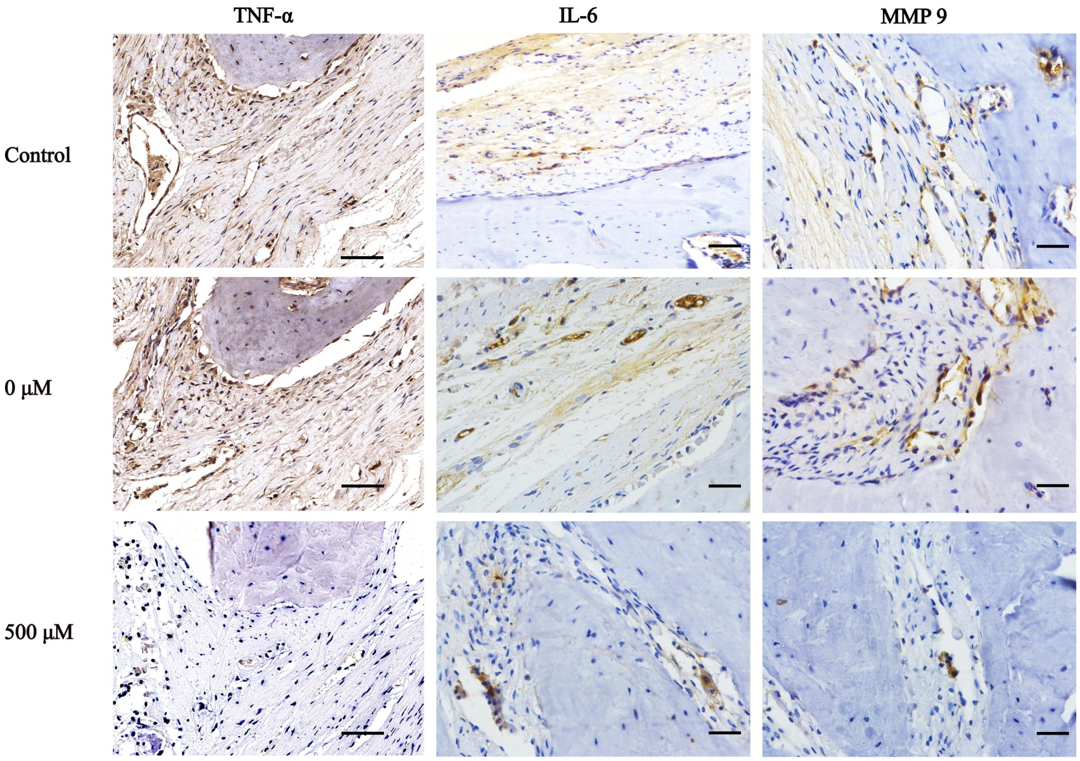

B

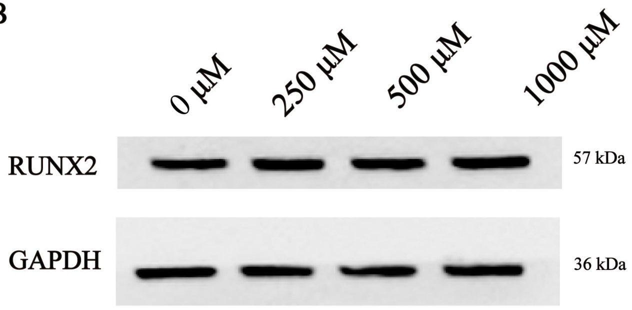

C

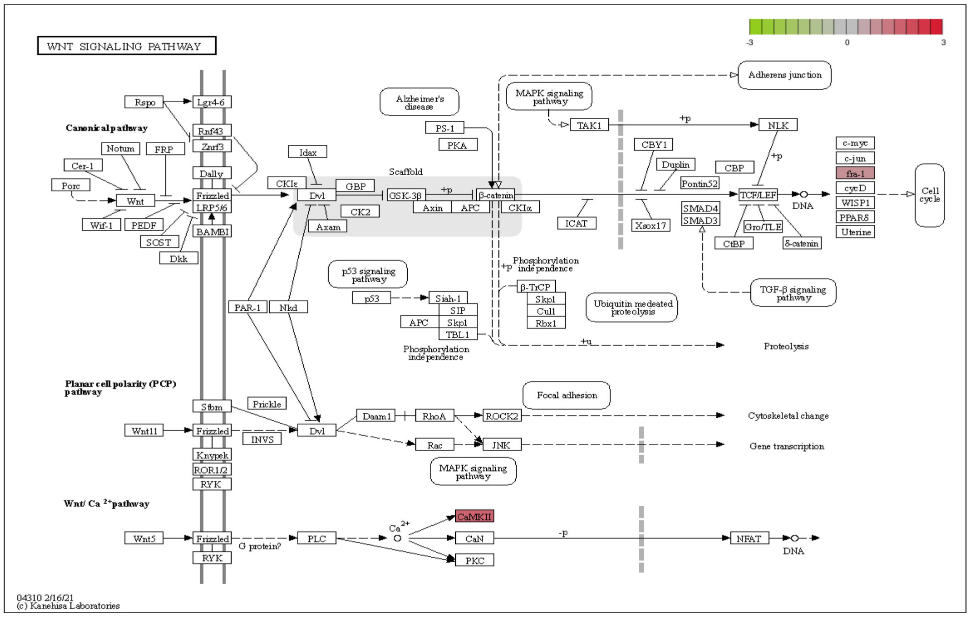

Supplementary Figure 2. Met/α-CSH/nHA attenuates the inflammatory response and KEGG analysis. Immunohistochemical staining results reveal a decrease in the expression of TNF-α, IL-6, and MMP9 containing 500 μM Met/α-CSH/nHA (A) (Scale bar: 50 μm). However, 500 μM Met/α-CSH/nHA induced osteogenesis in MC3T3-E1 cells that were not evident at 7 d (B); moreover, KEGG enrichment analysis revealed that the bone repair ability of Met/α-CSH/nHA material might be related to the Wnt signaling pathway.
